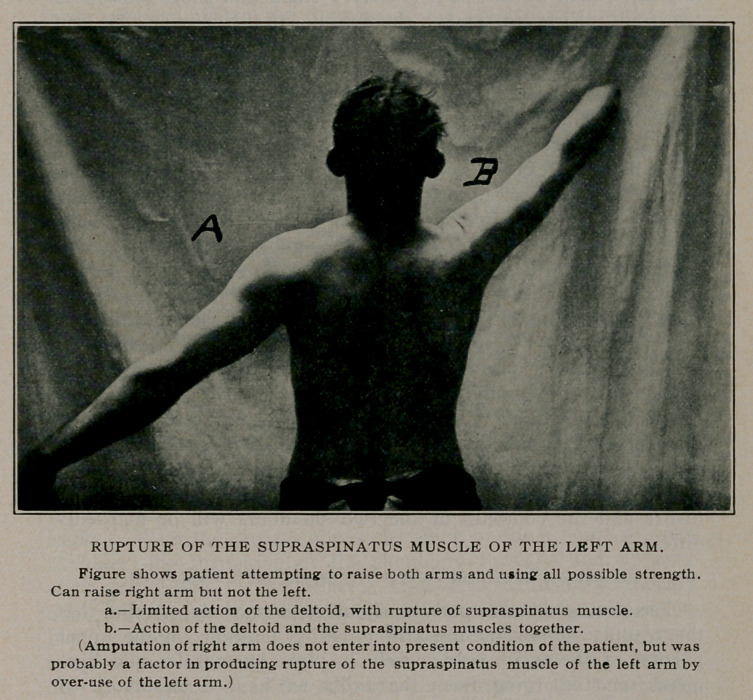# Some Orthopedic Conditions in the Neighborhood of the Shoulder Joint

**Published:** 1914-02

**Authors:** Roland O. Meisenbach

**Affiliations:** Buffalo, N. Y.


### Some Orthopedic Conditions in the Neighborhood of the Shoulder Joint

BY ROLAND O. MEISENBACH, M.D.
Buffalo, N. Y.

THE shoulder joint is usually injured in one of two ways: By direct force; that is, the shoulder coming in contact with some object, as is often the case in a fall onto the sidewalk or by indirect muscular action; that is, the person may suddenly bring the arm into play and cause a counter muscular resistance; as, for instance, in attempting to prevent a fall.

The shoulder joint is one of the joints of the body which is very frequently neglected, and sometimes when treated the best results are not obtained. This is, I believe, due chiefly to the fact that the shoulder joint is one of the most difficult joints to immobilize or to hold in the position which may be most favorable to one or the other forms of treatment. However, upon making the proper diagnosis, the results will be more pleasing than is generally supposed, if the condition is thoroughly understood.

In this paper I will not undertake to enumerate the many constitutional disturbances which may interfere with the shoulder joint motion as, for instance, in the hypertrophic, infectious, tuberculosis, or sarcomata and others, but wish to emphasize those which may follow injury, even though the injury is not a pronounced one, but may encumber the individual many months after the injury. This annoyance may manifest itself in usually one or two ways: either a full, active motion is not possible, or there may be a constant or an intermittent pain in the chest or down the arm, sometimes localizing in the shoulder joint itself. My attention has been called to a number of striking cases, in which relief was offered, in some instances by means of simple procedures and in others by operation. One of the most common injuries to the shoulder is the injury to the subdeltoid or acromion bursa.

### INJURY TO THE SUBDELTOID BURSA.

The subdeltoid bursa may often be the seat of injury following cases of dislocation or fracture in which the dislocation or fracture has been adjusted satisfactorily, but in which the patient complains of pain on pressure over the area of the bursa or in certain motions of the arm. Anatomically, the bursa varies considerably, sometimes very small and again very large, so that when injured its function is not properly maintained and the mechanism of the shoulder is naturally impaired. This is especially true when there is a predisposing diathesis. When the subdeltoid bursa is involved, a person is usually able to raise the arm over the head and in many directions, but the motion is painful, as the two walls of the bursa are usually adherent and may be thickened so that when the person raises the arm beyond the shoulder the bursa has difficulty in passing under the acromion process. In locating this bursa, and especially in subdeltoid bursitis, Dawbarn's sign is very helpful. With the arm hanging down, pressure over the site of the bursa will elicit pain, whereas, when the arm is raised up to the level of the shoulder, pressure over the same anatomical landmark will not elicit pain, the bursa having glided under the acromion process by the elevation of the arm. In fracture or dislocation of the shoulder the bursa may be injured and convalescence may be retarded to many months if the condition is not observed, and the retardation may be credited to an improper replacement of the original injury; whereas, in reality, there is simply a complication present.

RUPTURE OF THE SUPRASPINATUS MUSCLE.

In the subdeltoid bursitis injury by direct violence is usually the cause whereas, in rupture of the supraspinatus muscle indirect muscular action is the common cause. The function of the deltoid is to elevate the arm almost to the level of the shoulder, after which the supraspinatus comes into play and assists in raising the arm higher up. The function of the supraspinatus muscle, which, by the way, is a very small and short muscle, is two-fold. First, to remove the capsule of the joint as the arm is raised so that the head of the humerus will not impinge upon the capsule between the acromion process, and secondly, to assist the deltoid in elevating the arm above the shoulder. In some individuals this muscle is well developed; whereas in others it is only a few fibres and is difficult to find, especially when there is much bleeding, or if the injury is of a number of months standing. Whenever the supraspinatus muscle is ruptured, completely or partially, the individual is unable to lift the arm above the level of the shoulder, no matter how much effort is used. In some cases the diagnosis may be easily passed off as hysterical joint, but it is not easy to understand why a laborer with a large family, who is unable to work although well-muscled and healthy otherwise, will be unable to bring his arm above his shoulder, as in a case which came under my care. It is also not uncommon to see in the same individual a bursitis as well as a rupture of the supraspinatus muscle. This is especially true in the severer types of injury. The outside appearance of the shoulder joint in rupture of the supraspinatus muscle may be quite normal, but if of long standing, the deltoid will become somewhat atrophied by disuse and the contours of the two shoulders will be markedly different.

BRACHIAL PRESSURE WITH NEURITIS AS A SYMPTOM.

Considering the arm and the shoulder as a whole, we find that the scapula supporting the arm is really a saddle on the upper portion of the torso, held loosely in place by muscles and ligaments, and emerging from the axilla we have a meshwork of major nerves, arteries and veins passing in front and beneath this saddle, which radiate down the arm. In very heavy individuals the weight of an arm is considerable. If the individual is inactive or has a sedentary occupation much of this weight is borne by the saddle, with consequent pressure upon the structures beneath, chiefly the brachial plexus. The pressure will be manifested chiefly by pain in the periphery, so that when certain motions of the hand and fingers are made the arm will be painful. Usually in these people there is a tendency toward forward-stooped shoulders, and this aggravates the condition.

The weight of the arm is pulling the shoulders forward and downward, which increases the pressure upon the brachial plexus.

### REFERRED PAIN TO THE NEIGHBORHOOD OF THE SHOULDER DUE TO SLIGHTLY DEFORMED SCAPULA.

If one carefully examines anatomical specimens of scapulæ, both of children and adults, one is impressed immediately by three entities: First, the thinness of the scapula in children, in whom it is almost translucent; second, the variation in the superior border or tip; and third, the difference in the superior angle which rests upon the ribs.

In again considering the shoulder as a saddle, we find that we have two groups of muscles, the superior and the inferior, which oppose each other in the function of holding the shoulders erect and supporting the weight of the arm. In the scapula of the normal child the tip of the scapula will be smooth and bent backward, so that when active motion of the shoulder is attempted the scapula will glide easily over the ribs. On the other hand, if by virtue of the stoop shoulder the child is allowed to grow up in this way, there may develop a sharp-pointed scapula which will not glide as easily, so that when the child attempts an occupation there may be an irritation at the point of the scapula, with referred pain to the shoulder joint or, as in a few cases, to the anterior portion of the chest. This condition may be found in otherwise healthy appearing people, but upon examination it is found that the shoulders are forward, and upon deep palpation with the thumbs the angles may be felt in some cases to be forward. The sharpness, however, cannot be determined absolutely. With the thumbs above the spine of the scapula of each shoulder, the shrugging of the shoulders forward and up may elicit crepitation in the affected shoulder. It usually appears bilaterally, but in some instances in my experience the crepitation has appeared unilaterally. This is especially true when the chief cause is that of occupation; as, for instance, throwing a switch. The chief symptom is the subjective symptom of pain, but may be a pain which is not definitely localized, but which may involve the upper portion of the trunk and may radiate to the forward portion of the chest. The motions of the shoulder, as a rule, are not impaired, but seem freer than normal. The relief of this condition naturally suggests itself in two ways: one, and especially in children, whose scalpulæ can be easily molded, is to correct the posture by means of a back brace or otherwise. In the adult, where the condition warrants, operative interference gives permanent relief. The cases herewith cited are those of the various types which have come under my observation:

1. INJURY TO THE SUBDELTOID BURSA.

*Case I.* Mr. D., about forty years old, fell on the sidewalk, injuring his shoulder. Up to that time he had been actively at work, using the arms normally. After the injury, which occurred several months before he consulted me, he had been unable to work, owing to the fact that he could not raise his left arm, and it seemed to be gradually growing stiff. Examination showed a swelling of his left arm which suggested fluctuation in the region of the deltoid bursa. On deep palpation over the area of the bursa, patient would jump. After a careful study of the case, patient was sent to the hospital and the arm manipulated. A few months later patient returned to work.

2. RUPTURE OF THE SUPASPINATUS MUSCLE.

*Case II.* Mr. O., fifty-nine years old, referred to me June 29, 1909, by Dr. Lyon, with the following history: A year ago, while standing on the platform of a Pullman train, the coupling broke and the train started and the patient was deposited on the roadbed, throwing out both arms. He was taken to a hospital and examined and the chest strapped. He also sustained a sprain of the right ankle and fracture of two ribs, which yielded to treatment. He has been unable to use his right arm, some motions, especially raising the arm, being painful, and unable to get his arm above the level of the shoulder. Physical examination shows that the patient stands with right shoulder drooping downward and slightly forward. There is less fullness in the clavicular portion of the right shoulder than the left. Patient cannot get arm in abduction beyond level of shoulder without bringing the scapula into play; Dawbarn's sign present, slight crepitation present upon rotation; on rotating humerus inward, limitation of motion exists; external rotation with elbows at right angles limited. The X-ray showed that two ribs had been broken. Diagnosis: Rupture of the supraspinatus muscle, with a probable subdeltoid bursitis.

3. BRACHIAL PRESSURE WITH NEURITIS AS A SYMPTOM.

*Case III.* Rev. R., referred to me by Dr. Lyon, April, 1910, complained of pain down his arm, chiefly in his hand, while sitting at his desk. Patient unable to put on his coat or button it without pain, chiefly manifested in the hand, which would not yield to ordinary treatment. Diagnosis of brachial pressure made by Dr. Lyon, confirmed by myself. Physical examination showed contour of both shoulders equal, no special tenderness over bursa; active motion somewhat painful in certain directions. The attitude of the patient was somewhat toward stooped shoulders, the arms hanging forward. X-ray showed no general diathesis.

Stoop shoulders were corrected and the arm raised so that the weight was taken off the brachial plexus. Symptoms disappeared as long as the weight of the arm was removed from the plexus, but occurred as soon as the shoulder flap loosened. A webbing shoulder brace was then worn and the general tonicity of the shoulders gradually improved and poise and attitude of the patient changed so that after six weeks patient was able to go without apparatus. Symptoms have not recurred since.

4. REFERRED PAIN TO THE NEIGHBORHOOD OF THE SHOULDER DUE TO SLIGHTLY DEFORMED SCAPULA.

*Case IV.* Miss L., assistant in the office of a hospital, age 28, consulted me August 14, 1911, with a history of having pain in both hands, with general fatigue. Periodically for the last few years she had been treated by means of drugs, hydrotherapy, electricity and blue light by her family physician. The condition, fatigue and pain in the hands grew worse, especially while sitting on a high office chair at a high desk. Examination showed a very well-muscled, heavily-built young woman, good color and rather tall, standing with drooped shoulders and bent forward slightly, all neck muscles rather tense. Forward shrugging of shoulders elicited crepitation, especially noted on left side, but also some on right. Crepitation present in active and passive motion, angles of scapula forward and deep seated. Operation was performed August 16th, by exposing both angles of the scapula, which were found to be very sharp, so much so that they punctured my rubber gloves. The sharp angles were removed and the shoulders held in position during convalescence. Complete recovery; symptoms disappearing in two weeks.

Mr. F., a switchman, referred to me by Dr. Lyon, with the following history: Whenever he threw the switch, which he had to do many times a day, he felt referred pain in the forward part of his chest on the right side. Examination showed marked crepitation, which was transmitted over the entire shoulder blade when the patient shrugged his shoulders forward and upward. This was not obtained on the left side. Operation performed; angle of the scapula removed and the patient placed in a back brace. Patient able to return to his work without symptoms after a few months.

DIFFERENTIAL DIAGNOSIS.

It is true that one or more of the above conditions may occur in the same patient at the same time, but when occuring separately there are usually enough signs and symptoms that will lead to the correct diagnosis. The chief point of differentiation lies between the injury to the subdeltoid bursa and the rupture of the supraspinatus muscle. In the case of the subdeltoid bursitis, Dawbarn's sign, together, perhaps, with fluctuation and with the ability of the patient to passively but slightly move the arm in all directions, although painful, will be noted; whereas, in rupture of the supraspinatus there is usually atrophy of the deltoid due to disuse, the pain is not as great as in subdeltoid bursitis, but the arm cannot be brought beyond the level of the shoulder voluntarily, but can passively be placed there. If pain occurs in rupture of the supraspinatus muscle, it is usually of a sharp nature due to the impingement of the capsule, whereas in subdeltoid bursitis it occurs when the arm is moved in all directions.

In the differentiation between brachial pressure and deformity of the scapula, one must keep in mind that neuritis may occur as a symptom in both, and that one must distinguish between a true neuritis and neuritis as a symptom. Occasionally, in brachial pressure an absolute diagnosis cannot be made without instituting treatment; that is, removing the pressure, when the pain should shortly disappear. In deformity of the capsule, the crepitation upon forward flexion and elevation of the shoulders is almost pathognomonic, but may not necessarily determine the degree of deformity nor the shortness of the angle of the scapula, especially in adults. Where the stoop shoulders and the condition has persisted for a long time, giving only intermittent symptoms, there is usually a considerable amount of scar tissue formed, which accounts for some of the cases which have had shoulder symptoms at a previous time. The X-ray, in determining the diagnosis, may be of assistance in determining whether or not a general diathesis is present; as, for instance, when subdeltoid bursitis happens to become manifest in an individual who has already had hypertrophic arthritis. In this case the X-ray will show an outline of the bursa nicely, and if two plates are taken, one with the arm down and the other up, the change of position of the bursa will be noted. These types of case may often pass with a diagnosis of rheumatism, but do not yield after a thorough trial of the anti-rheumatic medications.

140 Allen Street.

---

Relation of Nerve Conductivity to Diameter. Lapique and Legendre, *Prog. Med.*, December 20, 1913, have found by experiments on the green frog that the average rapidity of conduction of nerve impulses vary directly as the diameter of the nerve fibres—in analogy with the law of electric resistance.

---

Danger of Benzol. E. Mühlman, *Deutsche Med. Woch-* No. 44, 1913. The author and Neumann have noted hepatic necrosis in one patient each, following the benzol treatment of myelogenic leucocythaemia. Similar results have followed experimental use of the drug in rabbits.

## Figures and Tables

**Figure f1:**